# Molecular Epidemiological Characteristics of Group A Rotavirus in Sika Deer in Jilin Province, China

**DOI:** 10.3390/vetsci13050452

**Published:** 2026-05-04

**Authors:** Yacong Li, Qilin Wang, Runlai Cao, Cheng Chang, Xiaoxu Wang, Zhijie Liu

**Affiliations:** 1 Key Laboratory of Special Animal Epidemic Disease, Ministry of Agriculture and Rural Affairs, Jilin Provincial International Cooperation Key Laboratory for Science and Technology Innovation of Special Animal and Plants, Institute of Special Animal and Plant Sciences, Chinese Academy of Agricultural Sciences, Changchun 130112, China; lycong910@163.com (Y.L.); wangqilin@caas.cn (Q.W.); caorunlai@caas.cn (R.C.); 821012430432@caas.cn (C.C.); 2 State Key Laboratory for Animal Disease Control and Prevention, Lanzhou Veterinary Research Institute, Chinese Academy of Agricultural Sciences, Lanzhou 730046, China

**Keywords:** sika deer, rotavirus, genotyping, epidemiological investigation

## Abstract

This study focuses on epidemiological characteristics and genetic evolution of rotavirus (RV) isolated from sika deer in Jilin Province, China. The objective is to determine the positivity rate and genotypes of rotavirus circulating in diarrheic sika deer fawns. Polymerase chain reaction (PCR) targeting the rotavirus VP6 gene was utilized to detect positive samples from the collected diarrheal samples. The positive samples were subjected to genotyping by amplification of the VP4 and VP7 genes, and then phylogenetic analysis was conducted to analyze the genetic diversity of the virus. As a result, 230 samples tested positive out of 438 diarrheic samples, resulting in a positivity rate of 52.5% in diarrheic samples. The genotyping analysis showed that the genotypes G6, G10, and G8 accounted for 70.59%, 26.47%, and 2.94%, respectively; while types P[1], P[11] and P[14] occupied 42.59%, 37.03% and 20.3%, respectively. Seven genotypic combinations were identified, with G6P[1] (34%) and G6P[11] (30%) being the most dominant. The successfully isolated SY-73 (G8P[1]) strain clustered with bovine RV strains, demonstrating the genetic diversity and recombination traits of the deer-derived RV. It was shown that the isolated SY-73 (G8P[1]) strain caused diarrhea in suckling mice, confirming its pathogenicity. Histopathological analysis further revealed intestinal lesions in infected mice, including villus atrophy and epithelial damage, supporting its in vivo pathogenic effects. This study indicates a relatively high infection rate of rotavirus in sika deer in the studied regions, characterized by complex and variable genotype combinations, with the potential for cross-species transmission.

## Simple Summary

Diarrhea is a common and serious health problem in young animals. Group A rotavirus is one of the most important pathogens causing diarrhea in sika deer fawns in the sampled regions of Jilin Province, the People’s Republic of China, which has a relatively high infection rate of rotavirus in sika deer. In this study, the detection rate of rotavirus in diarrheic sika deer fawns was 52.5%, and seven genotypic combinations were identified. The dominant genotypes were G6P[1] (34%) and G6P[11] (30%), and a G8P[1] strain was successfully isolated in vitro, and its pathogenicity was demonstrated in a mice model. Intestinal histopathological changes in infected mice further supported its pathogenic effects. These findings expand our understanding of rotavirus circulating in sika deer, which implies the necessity to implement control measures against virus transmission.

## 1. Introduction

Rotavirus (RV) is classified within the Reoviridae family and the Rotavirus genus [1]. It is a segmented dsRNA virus with a genome consisting of 11 segments. Rotaviruses are classified into 10 categories (A–J) based on variations in the VP6 antigen [2], with Group A rotavirus (RVA) being the principal pathogen responsible for acute gastroenteritis in newborns and certain young animals [3]. This virus has a wide host range, infecting farmed mammals, including cattle, sheep, pigs, dogs, and cats, as well as poultry and wild species such as deer [4,5]. Consequently, based on the genotypes of its capsid proteins VP7 and VP4, the academic community predominantly employs the G/P dual nomenclature system for genotyping, which has emerged as the principal standard for characterizing variation in RVA [6,7].

Epidemiological investigations and genotyping of RVA across many animal species have become increasingly significant in recent years. The prevalence of RVA in Korean water deer is 2% [4], while Slovenian roe deer are linked to the G8P[14]-type RVA. Phylogenetic investigations reveal that this viral strain exhibits genetic similarities to rotaviruses seen in cattle and humans [5,8]. In Germany, the G10P[15] genotype has been reported as a predominant strain in wild ruminants and exhibits high genetic homology with strains from roe deer, sheep, and cattle [9]. Furthermore, studies in the highland areas of Peru have recorded the co-infection of llamas, sheep, and people with multiple RVA genotypes, suggesting a possible danger of cross-species transmission [10]. Matthijnssens et al. performed a trace-back study of G6P[14] rotaviruses from various host sources, revealing significant whole-genome phylogenetic relatedness between human G6P[14] rotavirus strains and analogous strains from sheep and other even-toed ungulates [11]. Overall, these studies highlight the global diversity of RVA in different host species and regions, as well as its potential for interspecies transmission.

In China, research on RVA has mainly focused on livestock, particularly cattle and sheep. In calves, RVA infection rates can reach up to 58.3%, with G6P[1] identified as the predominant genotype [12,13]. In sheep, the prevalence of RVA ranges from 15.38% to 25%, with G6P[1] and G6P[11] being the dominant genotypes. In addition, ovine RVA strains exhibit high genetic similarity to bovine RVA in both genotype composition and genomic backbone [14,15]. In contrast, the epidemiological characteristics and genotype distribution of RVA in sika deer remain poorly understood.

Previous studies conducted in Jilin Province have shown that the etiological composition of diarrhea in sika deer fawns was found to be complex, with frequent co-infections involving multiple viral pathogens. Among these, coronavirus (59.44%), rotavirus (58.89%), and bovine viral diarrhea virus (21.67%) were identified as the predominant pathogens, and mixed infections were commonly observed [16]. Moreover, a rotavirus strain previously isolated in our study showed close genetic relatedness to bovine-origin viruses, suggesting the potential for cross-species transmission or host sharing within sika deer populations. These findings indicate that viral pathogens, particularly rotavirus, may play an important role in the occurrence of diarrhea in sika deer fawns. However, despite its relatively high detection frequency in diarrheic cases, the specific epidemiological characteristics, genotype distribution, and genetic evolution of RVA in diarrheic sika deer fawns remain limited. Therefore, focusing on clinically affected (diarrheic) individuals is essential for clarifying the potential role of rotavirus in disease occurrence and for understanding its molecular epidemiological features under field conditions.

Jilin Province is the main production area for sika deer farming in China, playing an important role in the supply of deer products [17]. China is one of the major producers of sika deer products worldwide, with Jilin Province serving as the core breeding and germplasm resource center. It is estimated that Jilin accounts for approximately 80% of the national sika deer output and over 50% of the national deer population, making it the leading supplier of deer products in China [18,19]. Fawn diarrhea has become a major disease restricting the healthy breeding and sustainable development of the sika deer industry, not only causing stunted growth in fawns but also leading to death in severe cases, resulting in significant economic losses for the breeding industry. Based on this, the present study investigates the infection rate, genotypes, and genetic characteristics of RVA in fawns within the sika deer population in the Jilin region. It also evaluates the potential risk of interspecies transmission, thereby providing a theoretical basis for the future prevention and control of deer diarrhea disease.

## 2. Materials and Methods

### 2.1. Ethical Approval

The samples were collected by licensed veterinarians and evaluated by the Animal Infectious Disease Prevention and Control Team of the Institute of Special Animal and Plant Sciences, Chinese Academy of Agricultural Sciences.

The People’s Republic of China’s Regulations for the Administration of Affairs Concerning Experimental Animals were followed in treating the laboratory animals. All experiments were approved by the Ethics Committee of the Institute of Special Animal and Plant Sciences, Chinese Academy of Agricultural Sciences (Approval No. ISAPSAEC-2025-020).

### 2.2. Collection and Processing of Fecal Samples

From June to September in both 2023 and 2024 (the peak period of diarrhea in sika deer), fecal samples from diarrheic fawns were collected from small-scale farms and four large-scale breeding farms in Shuangyang District, Changchun City, and Dongfeng County, Liaoyuan City, Jilin Province. A total of 438 fecal samples were collected. Defecation was induced by gentle anal stimulation, and fecal samples were immediately collected in sterile 50 mL centrifuge tubes to minimize contamination. All samples were stored at −80 °C until further analysis.

### 2.3. RNA Isolation

Fecal samples were resuspended in 0.9 mL PBS (pH 7.2) and vigorously mixed by vortexing for 5 min to ensure complete homogenization. The mixtures were clarified by centrifugation at 12,000 rpm for 10 min at 4 °C, then 200 μL of the clear supernatant was aseptically transferred into RNase-free microcentrifuge tubes for subsequent RNA isolation using the TransZol Up Plus RNA Kit (TransScript, Beijing, China).

For extraction, samples were processed with TransZol reagent to disrupt the fecal supernatant matrix and release viral RNA, followed by thorough mixing and centrifugation at 12,000 rpm for 15 min to separate phases. The upper phase was carefully collected and transferred to a new tube, then mixed with an equal volume of absolute ethanol to facilitate RNA binding, and immediately loaded onto a silica-based spin column. The column was sequentially rinsed with CB9 and WB9 wash buffers to remove impurities, followed by an additional centrifugation step to remove residual ethanol. Finally, RNA was eluted in 50 μL RNase-free water and preserved at −80 °C for downstream applications.

### 2.4. PCR Amplification

cDNA synthesis was performed using a One-Step gDNA Removal and cDNA Synthesis SuperMix kit (TransScript, Beijing, China). The VP6, VP4, and VP7 genes were amplified using 2× Taq Master Mix (Vazyme, Nanjing, China), while VP6 was used for detection and VP4/VP7 for G/P genotyping. Primers were designed using Primer 5.0 based on conserved regions (Table 1). PCR amplification was performed in a total reaction volume of 25 μL, consisting of 12.5 μL of 2× Taq Master Mix, 1 μL of each primer, 2 μL of cDNA template, and 8.5 μL of nuclease-free water. The thermocycling program began with an initial denaturation step at 95 °C for 5 min, followed by 35 amplification cycles, including denaturation at 95 °C for 30 s, annealing at 55 °C for 30 s, and extension at 72 °C for 1 min, and a final extension at 72 °C for 10 min. The amplified products were separated by electrophoresis on a 1% agarose gel and subsequently visualized using a gel documentation system [20].

### 2.5. Determination of VP7 and VP4 Gene Sequences and Phylogenetic Analysis

PCR products were purified using an E.Z.N.A. Gel Extraction Kit (Omega, Norcross, CA, USA) and cloned into the pMD18-T vector (Takara, Japan), followed by transformation into DH5α competent cells (TransScript, Beijing, China). Recombinant clones were selected on ampicillin-containing agar plates and incubated overnight at 37 °C. Positive clones identified by PCR were sequenced by Sangon Biotech (Shanghai, China) using the Sanger method. Reference rotavirus sequences were retrieved from the NCBI database. Sequence alignment was performed using SnapGene 8.2.1, and phylogenetic analysis was conducted using MEGA 11. Nucleotide similarities were calculated using the p-distance model, and phylogenetic trees were constructed using the neighbor-joining method based on the Kimura two-parameter model with 1000 bootstrap replicates to assess branch reliability [21,22].

### 2.6. Cell Cultures

African green monkey kidney cell line (Vero) cells were cultured in DMEM (Gibco, Shanghai, China) supplemented with 10% fetal bovine serum (FBS) (Gibco, Shanghai, China) and 1% penicillin–streptomycin (Solarbio, Beijing, China) [23]. The cells were passaged twice a week by adding 1 mL of 0.25% trypsin (Gibco, Shanghai, China), and after 2–3 min at 37 °C, trypsinization was terminated. Then, the cells were seeded into the medium at a ratio of 1:2, and additional medium was added to make a total of 5 mL. The cells were then placed in a 37 °C, 5% CO_2_ incubator for culture.

### 2.7. Viral Isolation and Identification

Fecal samples (1 g) from diarrheic fawns were homogenized in 5 mL phosphate-buffered saline (PBS), followed by centrifugation at 10,000 rpm for 5 min. The supernatant was collected and filtered through a 0.22 μm membrane (Merck Millipore, Darmstadt, Germany). The filtrate was treated with trypsin to a final concentration of 10 μg/mL and incubated at 37 °C for 1 h to activate the virus. Vero cells were seeded in T25 flasks and cultured until approximately 85% confluence was reached. After washing twice with serum-free DMEM, the cells were inoculated with the activated viral suspension and incubated at 37 °C with 5% CO_2_ for 60 min for virus adsorption (without centrifugation). The inoculum was then removed and replaced with serum-free DMEM containing 5 μg/mL trypsin, followed by continued incubation at 37 °C with 5% CO_2_ for 48–72 h until cytopathic effects (CPEs) were observed. At 72 h post-infection, cultures were harvested and subjected to three freeze–thaw cycles, followed by centrifugation at 2000 rpm for 10 min. The supernatant was collected and used for serial passaging, with each passage lasting 72 h [24].

### 2.8. Transmission Electron Microscopy (TEM)

Viral morphology was characterized by negative-staining transmission electron microscopy (TEM, Hitachi TME HT7800, Tokyo, Japan). The virus-containing supernatant was clarified by centrifugation at 12,000 rpm for 15 min at 4 °C, followed by filtration through a 0.22 μm membrane. A 5 μL aliquot of the filtrate was applied onto a carbon-coated copper grid (200 mesh) and allowed to adsorb for 2 min. Excess fluid was wicked off with filter paper. The grid was negatively stained with phosphotungstic acid (pH 6.8), air-dried at room temperature, and subsequently examined using a Hitachi HT7800 transmission electron microscope to visualize viral particles [25].

### 2.9. Viral Growth Curve

Vero cells were seeded in 24-well cell culture plates and infected with the virus when the cells reached 70% confluence, with a multiplicity of infection (MOI) of 0.01 [26]. After 1 h of infection, the cells were washed three times with DMEM without fetal bovine serum (FBS) and then cultured with DMEM containing 5 μg/mL trypsin and no FBS. At various time points (0 h, 8 h, 16 h, 24 h, 32 h, 40 h, 48 h, 56 h, and 64 h), viral supernatants and whole-cell lysates were collected from each well. All samples were stored at −80 °C for quick freezing. Viral titers at each time point were determined by the median tissue culture infectious dose (TCID_50_) assay. Briefly, samples collected at different time points were subjected to 10-fold serial dilutions and inoculated in growth kinetics Vero cells seeded in 96-well plates. After incubation at 37 °C with 5% CO_2_ for 72 h, cytopathic effects (CPEs) were observed under a microscope. The TCID_50_ values were calculated using the Reed–Muench method, and viral growth curves were constructed [27].

### 2.10. Pathogenicity of Strains

Specific pathogen-free Kunming suckling mice (*n* = 12, 7 days old) were randomly divided into an experimental group (oral inoculation with 0.1 mL containing 1.78 × 10^6^ TCID_50_ rotavirus particles) and a control group (0.1 mL DMEM), as previously described [28]. Suckling mice were monitored daily for clinical signs, including activity, feeding behavior, and diarrhea.

Intestinal and rectal tissues were collected and homogenized in DMEM at a 1:3 (*w*/*v*) ratio, followed by centrifugation at 8000 rpm for 10 min at 4 °C. The supernatant was collected for RNA extraction and cDNA synthesis, as described by Yan et al. [29]. PCR amplification was performed using VP6-specific primers, as shown in Table 1.

### 2.11. Histopathology Analysis

At 72 h post-infection with RVA strain SY-73, mice were humanely euthanized and small intestinal tissues were collected. Samples were fixed in 10% neutral buffered formalin, paraffin-embedded, sectioned at 4–5 μm, and stained with hematoxylin and eosin. Histopathological changes were examined under a light microscope at 100× magnification [30].

## 3. Results

### 3.1. Epidemiological Investigation

Among 438 diarrheic samples, 230 tested positive for rotavirus, yielding an overall positivity rate of 52.5%. In 2023, 212 of 360 samples were positive (58.9%), whereas in 2024, 18 of 78 samples were positive (23.1%). Regionally, the positivity rate in Shuangyang District decreased from 67.9% in 2023 to 31.8% in 2024, while in Dongfeng County it declined from 51.5% to 11.7% (Table 2). Sequence analysis of the VP6 gene confirmed that all positive samples belonged to group A rotavirus (RVA).

**Figure 1:**
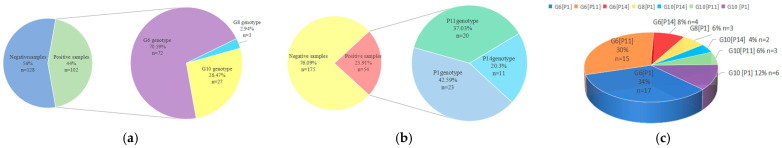
Distribution of RVA VP7 and VP4 genotyping and G/P genotype combinations. (**a**) Results of VP7 gene fragment genotyping; (**b**) Results of VP4 gene fragment genotyping; (**c**) Proportion of G/P genotype combinations of VP7 and VP4.

Among the 230 positive samples, VP7 genes were successfully amplified in 102 cases (44.34%), including G6 (70.59%), G10 (26.47%), and G8 (2.94%) (Figure 1a). VP4 genes were identified in 54 samples (23.91%), comprising P[1] (42.59%), P[11] (37.03%), and P[14] (20.30%) (Figure 1b). A total of seven G/P genotype combinations were identified, with G6P[1] (34%) and G6P[11] (30%) being predominant, followed by G10P[1] (12%), G6P[14] (8%), G8P[1] (6%), G10P[11] (6%), and G10P[14] (4%) (Figure 1c).

### 3.2. Phylogenetic Analysis of the VP7 Gene

**Figure 2:**
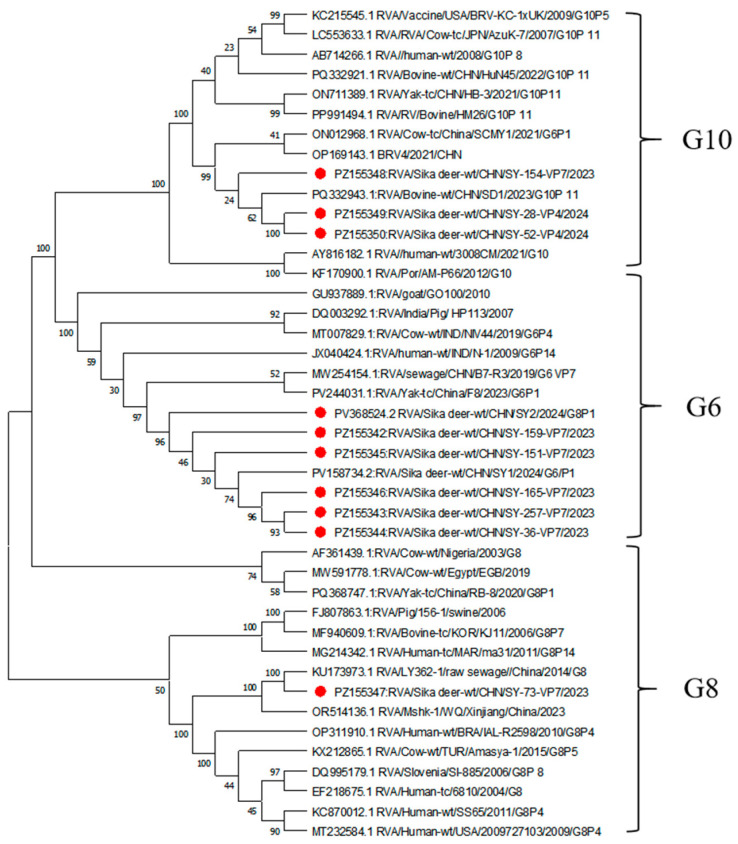
Phylogenetic tree based on nucleotide sequences of the VP7 gene of rotavirus A strains. The tree was constructed using the neighbor-joining method in MEGA 11, with evolutionary distances calculated using the p-distance model. Bootstrap values were determined from 1000 replicates. The reference sequences used in this study are listed in Table S1, and the sequences identified in this study (highlighted in red) are provided in Table S2.

VP7 gene analysis identified three G genotypes (G6, G8, and G10) among rotavirus-positive samples from sika deer. Representative sequences, including five G6 strains, two G8 strains, and three G10 strains, were selected for phylogenetic analysis(Figure 2). The G6 strains (SY-36, SY-165, SY-257, SY-159, and SY-151) clustered with a previously identified sika deer-derived strain (PV368524) from our laboratory with nucleotide identities ranging from 96.70 to 96.91%. The G8 strains (SY-2 and SY-73) were closely related to bovine strain OR514136 from Xinjiang Uygur Autonomous Region and an environmental strain KU173973, with nucleotide identities ranging from 95.17% to 96.70%. The G10 strains (SY-154, SY-52, and SY-28) clustered with a bovine strain (PQ332932) from Jiangsu Province, showing nucleotide identities of 87.43–97.74%.

### 3.3. Phylogenetic Analysis of the VP4 Gene

**Figure 3:**
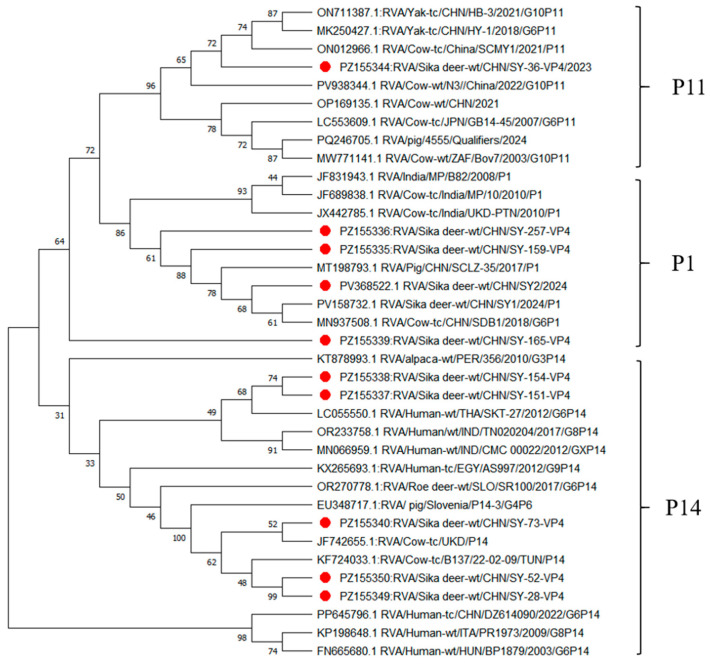
Phylogenetic tree based on nucleotide sequences of the VP4 gene of rotavirus A strains. The tree was constructed using the neighbor-joining method in MEGA 11, with evolutionary distances calculated using the p-distance model. Bootstrap values were estimated from 1000 replicates. The reference sequences used in this study are listed in Table S1, and the sequences identified in this study (highlighted in red) are provided in Table S2.

VP4 gene analysis identified three P genotypes (P[1], P[11], and P[14]) among rotavirus-positive samples from sika deer. Representative sequences, including five P[1] strains, two P[11] strains, and three P[14] strains, were selected for phylogenetic analysis(Figure 3). The P[1] strains (SY-28, SY-159, SY-257, SY-2, and SY-73) clustered with a bovine rotavirus strain (MN937507), showing nucleotide identities of 85.36–97.56%. The P[11] strains (SY-36 and SY-52) were closely related to a yak-derived strain (ON711387), with nucleotide identities of 96.26–97.79%. Notably, the P[14] strains (SY-165, SY-151, and SY-154) clustered with a human-derived strain from Thailand (LC055550), showing high nucleotide identities of 96.56–97.99%, suggesting a close genetic relationship between deer- and human-derived strains.

### 3.4. Isolation and Identification of Rotavirus

**Figure 4:**
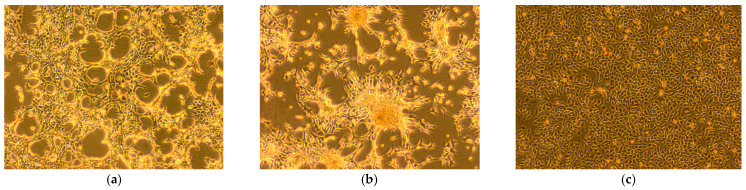
Virus isolation (40× magnification). (**a**) Vero cells infected with SY-73 virus for 24 h; (**b**) Vero cells infected with SY-73 virus for 48 h; (**c**) control group Vero cells.

PCR-positive samples were processed and used to infect Vero cells for virus isolation. Cytopathic effects were observed within 24 h post-infection, characterized by cell rounding, shrinkage, narrowing of cell margins, and aggregation (Figure 4a). At 48 h post-infection, cell detachment and a network-like cytopathic pattern were observed, whereas control cells remained normal (Figure 4b,c). The isolated virus was identified as rotavirus and designated as RVA/sika deer-wt/SY-73/2023 (SY-73).

### 3.5. Electron Microscopy

**Figure 5:**
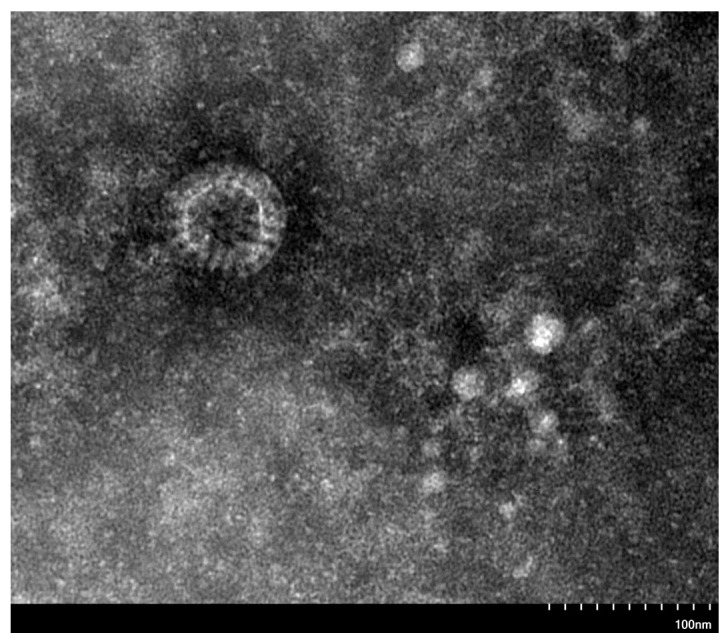
Transmission electron micrograph of rotavirus particles (strain SY-73) at 25,000× magnification.

Following concentration and purification of the 10th passage (P10) viral culture of strain SY-73, virus particles exhibited a typical spherical morphology with a well-defined double-layered capsid. Radially arranged spike-like structures were observed between the capsid layers, forming the characteristic wheel-like appearance of rotavirus. The particle diameter was approximately 70 nm (Figure 5).

### 3.6. Viral Growth Kinetics

Vero cells were infected with the SY-73 strain at a multiplicity of infection (MOI) of 0.01, and viral titers were quantified at the indicated time points (0–64 h post-infection) to characterize the viral growth kinetics.

**Figure 6:**
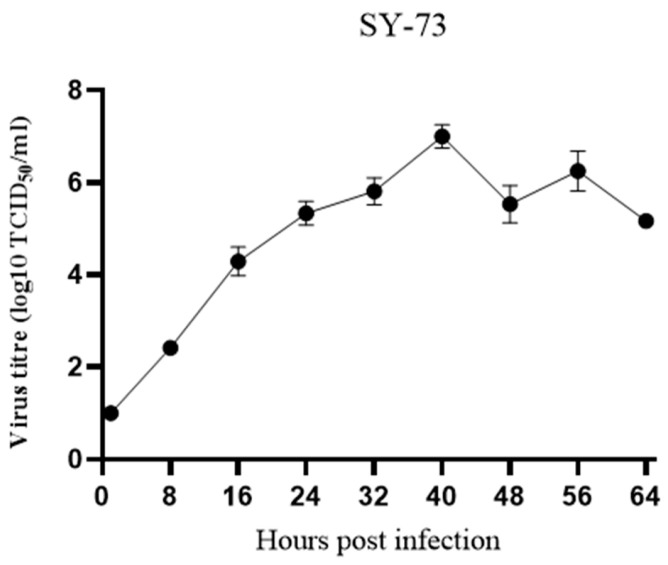
SY-73 virus growth curve.

Vero cells were infected with the SY-73 strain at a multiplicity of infection (MOI) of 0.01. Viral titers were determined at 0, 8, 16, 24, 32, 40, 48, 56, and 64 h post-infection to generate a growth curve. The virus exhibited rapid replication starting at 8 h post-infection, with the titer peaking at approximately 40 h, followed by a slight decline and stabilization at a relatively constant level (Figure 6).

### 3.7. Pathogenicity of the SY-73 Strain in Suckling Mice

**Figure 7:**
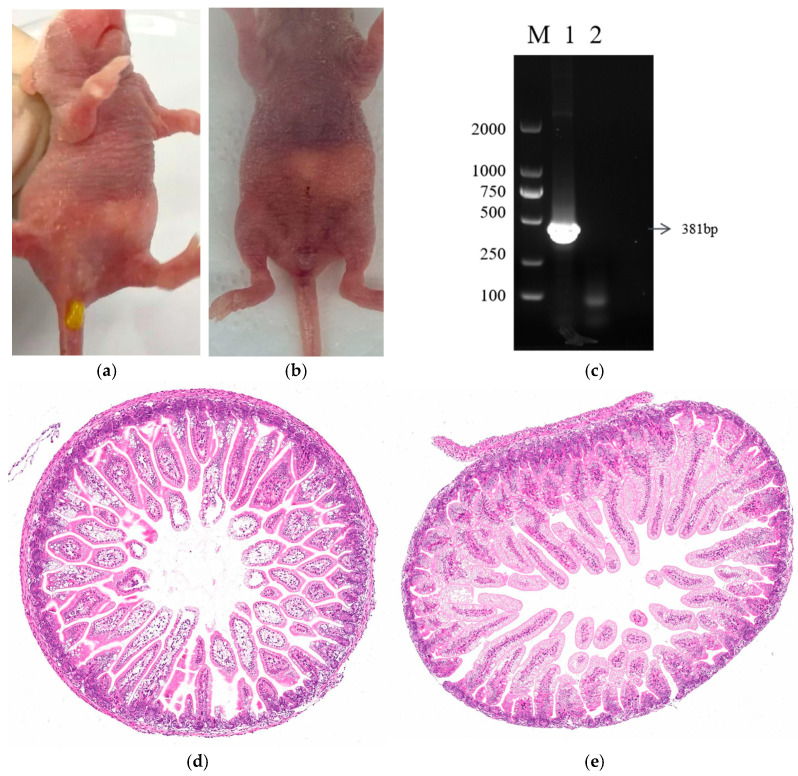
Pathogenicity of the SY-73 strain in suckling mice. (**a**) Mice infected with SY-73; (**b**) control mice; (**c**) PCR detection of the VP6 gene in intestinal tissues, where lane 1 represents infected mice, lane 2 represents control mice, and M indicates the 2000 bp DNA marker; (**d**) small intestine tissues of the control suckling mice had normally arranged villi (100×); (**e**) the small intestinal tissues of SY-73–infected suckling mice showed villus shortening and shedding (100×) (the original PCR pictures can be found in Figure S1).

Mice infected with the RVA SY-73 strain developed diarrhea, characterized by soft or watery pale yellow feces (Figure 7a). No clinical signs, including diarrhea or mortality, were observed in the control group (Figure 7b). Intestinal tissues were collected for RNA extraction and PCR analysis, and the results confirmed the presence of the VP6 gene with the expected fragment size (Figure 7c). Pathological alterations in the small intestine were evaluated in mice infected with RVA strain SY-73 at 72 h post infection. The control group exhibited normal intestinal morphology, with intact and well-organized villi. In contrast, infected mice showed pronounced histopathological changes, including villus atrophy, epithelial cell shedding, and disruption of villus architecture (Figure 7d,e).

## 4. Discussion

This study investigated rotavirus infection in diarrheic sika deer from large-scale farms and smallholder systems in Dongfeng County and Shuangyang District, Jilin Province. The overall positivity rate of 52.5% indicates that rotavirus is likely an important contributor to diarrhea in fawns in this region. Stratified analysis revealed higher infection rates in smallholder systems compared with large-scale farms in both regions, suggesting that differences in management practices and biosecurity measures may influence viral transmission. In addition, Shuangyang District, as a major trading center for sika deer products in China, may present increased transmission risks due to high animal movement and challenges in standardized management [31]. Previous studies have reported varying rotavirus prevalence across species, including alpacas (20.3%), llamas (47.5%), sheep (100%), and humans (33.3%) in high-altitude environments, with frequent co-infections observed [10]. In northeastern China, the prevalence of rotavirus in cattle was reported to be 58.3% [32], which is numerically similar to the findings in sika deer in the present study. However, it should be noted that the samples analyzed in this study were obtained from animals with diarrhea, which may lead to a higher detection rate of rotavirus. In contrast, the detection rates of rotavirus in wild animals are markedly lower, including 2% in Korean water deer [4], 2.1% in wild animals in Germany [9], and 1.0% in roe deer in Slovenia [5,8]. Therefore, differences in sampling strategies and clinical status should be taken into account when comparing prevalence rates across studies. These data are substantially lower than those observed in farmed deer, suggesting fundamental differences in epidemiological patterns and transmission dynamics between domestic and wild animals. Domesticated animals have more frequent contact with humans, and such interactions may alter transmission routes and epidemiological patterns, thereby increasing the potential for cross-species transmission and zoonotic events of rotavirus. These findings provide preliminary insights for future epidemiological surveillance and the prevention and control of rotavirus infections in the studied regions.

Genotyping analysis revealed that G6 (70.59%) and P[1] (42.59%) were the predominant genotypes of RVA in sika deer from Jilin Province, with seven G/P genotype combinations identified, including G8P[1], G6P[1], G10P[1], G6P[11], G6P[14], G10P[14], and G10P[11]. Notably, G6, G10, P[1], and P[11] are among the most common genotypes circulating in ruminants such as cattle and sheep worldwide [12,33,34,35]. The high prevalence of G6P[1] and G6P[11], which are dominant combinations in calves in China [13,36,37], further supports a close genetic relationship between deer and other ruminants. Phylogenetic analysis demonstrated that certain strains clustered with a human-derived rotavirus strain (LC055550), showing high nucleotide identities (96.6–98.0%). Previous studies have suggested that human P[14] strains may originate from interspecies transmission between humans and artiodactyls [37], with examples such as PR1300 and PR1973 in Italy being linked to artiodactyl hosts [12]. In this study, the clustering of P[14] sequences with human strains, together with the detection of G6P[14] and G10P[14] combinations, provides further evidence of potential genetic exchange among deer, cattle, and humans. These findings suggest the potential risk of cross-species transmission of rotavirus in these studied regions and provide useful information for further investigation of its possible public health implications, as well as challenges for future vaccine development and disease control strategies.

In this study, a G8P[1] genotype RVA strain, designated as SY-73, was successfully isolated from diarrheic fecal samples of sika deer. Phylogenetic analyses based on the VP7 and VP4 genes revealed that the G8 segment of SY-73 clustered with a bovine-derived strain from Xinjiang (OR514136), while the P[1] segment grouped within the same evolutionary branch as a bovine strain (MN937507), suggesting that this strain may have originated from a natural reassortment between bovine and deer RVA strains. Previous metagenomic studies have demonstrated the presence of multiple viral co-infections in diarrheic sika deer, with several rotavirus gene segments showing high similarity to bovine- and human-derived strains and clustering across different host species [16]. These findings indicate that rotaviruses may undergo genetic exchange among different hosts through reassortment. Consistent with these observations, our results further support the potential occurrence of cross-species transmission or inter-host viral sharing within sika deer populations. Given the close contact between sika deer, livestock (such as cattle), and humans during farming practices, viruses may potentially be transmitted between species through direct contact or shared environmental sources [38]. In addition, animal experiments demonstrated that the SY-73 strain induced typical diarrheal symptoms in 7-day-old suckling mice, as evidenced by typical diarrheal symptoms and the detection of the viral VP6 gene in intestinal tissues. Histopathological examination further revealed evident intestinal lesions in infected mice, including villous atrophy, epithelial shedding, and disruption of villous architecture, indicating that SY-73 infection can induce structural damage to the intestinal mucosa. When these findings are combined with viral RNA detection in fecal samples, they provide complementary evidence supporting active viral replication and in vivo pathogenic effects of this strain. These observations are consistent with previous studies showing that rotavirus infection in neonatal mice leads to diarrhea and intestinal damage, such as the study by Rey et al., in which oral inoculation of 7-day-old BALB/c mice resulted in persistent mild diarrhea at 3–4 days post-infection [31]. Similar approaches for virus isolation and characterization have also been widely applied in rotavirus research [37]. The successful isolation and pathogenic characterization of the SY-73 strain provide an important viral resource for further investigation of the pathogenic mechanisms of deer-derived RVA and for future vaccine development.

In summary, this study provides an initial investigation of rotavirus infection in sika deer in selected regions of Jilin Province, revealing a relatively high positivity rate (52.5%) in the sampled populations, with G6 identified as the predominant genotype. A total of seven G/P genotype combinations were identified, indicating considerable genetic diversity. Notably, phylogenetic analysis showed that P[14] genotype strains clustered with human-derived rotavirus strains, while the G8P[1] isolate exhibited close genetic relatedness to bovine strains, providing molecular epidemiological evidence suggesting the possibility of cross-species transmission and potential zoonotic risk of deer-derived rotavirus. These findings contribute to the limited available data on rotavirus in sika deer in China and provide a foundation for further studies on transmission dynamics between ruminants and humans, as well as for the development of effective prevention and control strategies.

## Figures and Tables

**Table 1:** RV-specific primers.

Primer	Nucleotide Sequence (5′–3′)	Product
VP6-F	CGAAGGCACNTTATACTCCA	391
VP6-R	ACCCGTTCTTTGTGTNCTAT	
VP4-F	TGGAAAGAAATGCAATACAATAG	818
VP4-R	ATTCCTGAAAACATTGAAAACAT	
VP7-F	GGCTTTAAAAGNGAGAATTTCC	1062
VP7-R	GGTCACATCATACANNTCTAAT	

**Table 2:** The positive rate of rotavirus in each region.

Region	Time	Farm		Free-Range Farmer	
		Positive/No. of Animals	Prevalence %	Positive/No. of Animals	Prevalence %
Shuangyang District	2023	29/47	61.7	81/115	70.43
	2024	4/15	26.47	10/29	34.48
Dongfeng County	2023	24/57	42.11	78/141	55.31
	2024	2/10	20	2/24	8.3

## Data Availability

The genomic sequences obtained in this study have been deposited in the NCBI GenBank repository under the accession numbers provided directly within the manuscript.

## Supplementary Materials

The following supporting information can be downloaded at: https://www.mdpi.com/article/10.3390/vetsci13050452/s1. Figure S1: Original image of PCR; Table S1: Reference sequences used for the construction of phylogenetic trees. Table S2: Information of virus sequence information in this study.

## Abbreviations

The following abbreviations are used in this manuscript:

RV	Rotavirus
RVA	Rotavirus A
PCR	Polymerase chain reaction
PBS	Phosphorus-buffered saline
Vero	African green monkey kidney cell line
FBS	Fetal bovine serum
