# Supplementary material for: Molecular Epidemiological Characteristics of Group A Rotavirus in Sika Deer in Jilin Province, China
Source: Vet Sci. 2026 May 4;13(5):452. doi: 10.3390/vetsci13050452 (PMC13211370; doi:10.3390/vetsci13050452)
Supplement: Supplementary file 1 [file vetsci-13-00452-s001.zip › Supplementary table.pdf]

Table S1. Reference sequences used for the construction of phylogenitical trees.

Primer	Description	GenBank ID	Year	genotype
VP4	RVA/Cow-wt/N3//China	PV938344.1	2022	P11
VP4	RVA/pig/4555/Qualifiers	PQ246705.1	2024	P11
VP4	RVA/Sika deer-wt/CHN/SY1	PV158732.1	2024	P1
VP4	RVA/Human-tc/CHN/DZ614090	PP645796.1	2022	P14
VP4	RVA/Roe deer-wt/SLO/SR100	OR270778.1	2017	P14
VP4	RVA/Human/wt/IND/TN020204	OR233758.1	2017	P14
VP4	RVA/Cow-wt/CHN	OP169135.1	2021	P11
VP4	RVA/Yak-tc/CHN/HB-3	ON711387.1	2021	P11
VP4	RVA/Cow-tc/China/SCMY1	ON012966.1	2021	P11
VP4	RVA/Cow-wt/ZAF/Bov7	MW771141.1	2003	P11
VP4	RVA/Pig/CHN/SCLZ-35	MT198793.1	2017	P1
VP4	RVA/Cow-tc/CHN/SDB1	MN937508.1	2018	P1
VP4	RVA/Human-wt/IND/CMC 00022	MN066959.1	2012	P14
VP4	RVA/Yak-tc/CHN/HY-1	MK250427.1	2018	P11
VP4	RVA/Cow-tc/JPN/GB14-45	LC553609.1	2007	P11
VP4	RVA/Human-wt/THA/SKT-27	LC055550.1	2012	P14
VP4	RVA/Human-tc/EGY/AS997	KX265693.1	2012	P14
VP4	RVA/alpaca-wt/PER/356	KT878993.1	2010	P14
VP4	RVA/Human-wt/ITA/PR1973	KP198648.1	2009	P14
VP4	RVA/Cow-tc/India/UKD-PTN	JX442785.1	2010	P1
VP4	RVA/India/MP/B82	JF831943.1	2008	P1
VP4	RVA/Cow-tc/India/MP/10	JF689838.1	2010	P1
VP4	RVA/Human-wt/HUN/BP1879	FN665680.1	2003	P14
VP4	RVA/ pig/Slovenia/P14-3/G4P6	EU348717.1	2008	P14
VP4	RVA/Cow-tc/UKD	JF742655.1	2009	P14
VP4	:RVA/Cow-tc/B137/22-02-09/TUN	KF724033.1	2014	P14
VP7	RVA//human-wt	AB714266.1	2008	G10
VP7	RVA/Cow-wt/Nigeria	AF361439.1	2003	G8
VP7	RVA//human-wt/3008CM	AY816182.1	2021	G10
VP7	RVA/India/Pig/ HP113	DQ003292.1	2007	G6
VP7	RVA/Slovenia/SI-885	DQ995179.1	2006	G8
VP7	RVA/Pig/156-1/swine	FJ807863.1	2006	G8
VP7	RVA/Human-tc/6810	EF218675.1	2004	G8
VP7	RVA/goat/GO100	GU937889.1	2010	G6
VP7	RVA/human-wt/IND/N-1	JX040424.1:	2009	G6
VP7	RVA/Vaccine/USA/BRV-KC-1xUK	KC215545.1	2009	G10
VP7	RVA/Human-wt/SS65	KC870012.1	2011	G8
VP7	RVA/Por/AM-P66/2012	KF170900.1	2012	G10
VP7	RVA/LY362-1/raw sewage//China	KU173973.1	2014	G8
VP7	RVA/Cow-wt/TUR/Amasya-1	KX212865.1	2015	G8
VP7	RVA/RVA/Cow-tc/JPN/AzuK-7	LC553633.1	2007	G10
VP7	RVA/Bovine-tc/KOR/KJ11	MF940609.1	2006	G8
VP7	RVA/Human-tc/MAR/ma31	MG214342.1	2011	G8
VP7	RVA/Cow-wt/IND/NIV44	MT007829.1	2019	G6
VP7	RVA/Human-wt/USA/2009727103	MT232584.1	2009	G8
VP7	RVA/sewage/CHN/B7-R3	MW254154.1	2019	G6
VP7	RVA/Cow-wt/Egypt/EGB	MW591778.1	2019	G8
VP7	RVA/Cow-tc/China/SCMY1	ON012968.1	2021	G6

VP7	RVA/Yak-tc/CHN/HB-3	ON711389.1	2021	G10
VP7	RVA/BRV4/CHN	OP169143.1	2021	G10
VP7	RVA/Human-wt/BRA/IAL-R2598	OP311910.1	2010	G8
VP7	RVA/Mshk-1/WQ/Xinjiang/China	OR514136.1	2023	G8
VP7	RVA/RV/Bovine/HM26	PP991494.1	2024	G10
VP7	RVA/Bovine-wt/CHN/HuN45	PQ332921.1	2022	G10
VP7	RVA/Bovine-wt/CHN/SD1	PQ332943.1	2023	G10
VP7	RVA/Yak-tc/China/RB-8	PQ368747.1	2020	G8
VP7	RVA/Sika deer-wt/CHN/SY1	PV158734.2	2024	G6
VP7	RVA/Yak-tc/China/F8	PV244031.1	2023	G6

Table S2. Information of virus sequence information in this study.

Primer	Description	GenBank ID	Year	genotype
VP4	RVA/Sika deer-wt/CHN/SY-159-VP4	PZ155335	2023	P1
VP4	RVA/Sika deer-wt/CHN/SY-257-VP4	PZ155336	2023	P1
VP4	RVA/Sika deer-wt/CHN/SY-151-VP4	PZ155337	2023	P14
VP4	RVA/Sika deer-wt/CHN/SY-154-VP4	PZ155338	2023	P14
VP4	RVA/Sika deer-wt/CHN/SY-165-VP4	PZ155339	2023	P14
VP4	RVA/Sika deer-wt/CHN/SY-73-VP4	PZ155340	2023	P1
VP4	RVA/Sika deer-wt/CHN/SY-36-VP4	PZ155341	2023	P11
VP4	RVA/Sika deer-wt/CHN/SY-2-VP4	PV368522	2023	P1
VP4	RVA/Sika deer-wt/CHN/SY-28-VP4	PZ155349	2024	P1
VP4	RVA/Sika deer-wt/CHN/SY-52-VP4	PZ155350	2024	P11
VP7	RVA/Sika deer-wt/CHN/SY-159-VP7	PZ155342	2023	G6
VP7	RVA/Sika deer-wt/CHN/SY-257-VP7	PZ155343	2023	G6
VP7	RVA/Sika deer-wt/CHN/SY-36-VP7	PZ155344	2023	G6
VP7	RVA/Sika deer-wt/CHN/SY-151-VP7	PZ155345	2023	G6
VP7	RVA/Sika deer-wt/CHN/SY-165-VP7	PZ155346	2023	G6
VP7	RVA/Sika deer-wt/CHN/SY-73-VP7	PZ155347	2023	G8
VP7	RVA/Sika deer-wt/CHN/SY-154-VP7	PZ155348	2023	G10
VP7	RVA/Sika deer-wt/CHN/SY-52-VP7	PZ155351	2023	G10
VP7	RVA/Sika deer-wt/CHN/SY-28-VP7	PZ155352	2024	G10
VP7	RVA/Sika deer-wt/CHN/SY-2-VP7	PV368524	2024	G8
