# Supplementary figures and images for: Molecular Epidemiological Characteristics of Group A Rotavirus in Sika Deer in Jilin Province, China
Source: Vet Sci. 2026 May 4;13(5):452. doi: 10.3390/vetsci13050452 (PMC13211370; doi:10.3390/vetsci13050452)

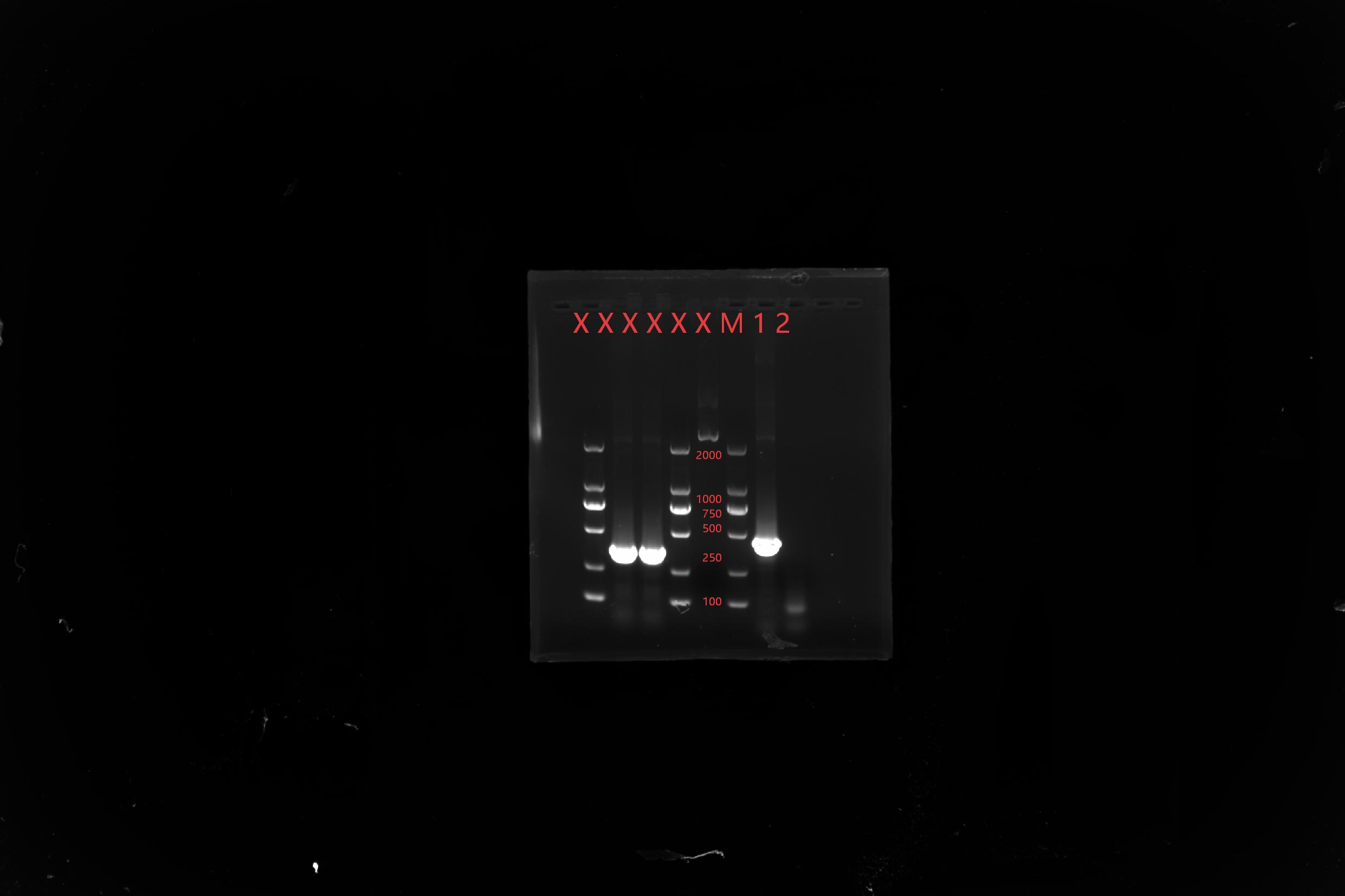

Supplement: Supplementary file 1 [file vetsci-13-00452-s001.zip › Electrophoresis original image.jpg]
